# Risk factors analysis and prediction model establishment of acute kidney injury after heart valve replacement in patients with normal renal function

**DOI:** 10.3389/fcvm.2025.1422870

**Published:** 2025-02-10

**Authors:** Xiaofan Huang, Xiangyu Sun, Jiangang Song, Yongqiang Wang, Jindong Liu, Yu Zhang

**Affiliations:** ^1^ Department of Anesthesiology, The Affiliated Hospital of Xuzhou Medical University, Xuzhou, Jiangsu, China; ^2^ Department of Anesthesiology, Shuguang Hospital Affiliated with Shanghai University of Traditional Chinese Medicine, Shanghai, China; ^3^ Jiangsu Province Key Laboratory of Anesthesiology, Xuzhou Medical University, Xuzhou, Jiangsu, China

**Keywords:** acute kidney injury, dynamic nomogram, heart valve replacement, normal renal function, prediction model

## Abstract

**Background:**

The study aimed to develop a risk prediction model through screening preoperative risk factors for acute kidney injury (AKI) after heart valve replacement in patients with normal renal function.

**Methods:**

A total of 608 patients with normal renal function who underwent heart valve replacement from November 2013 to June 2022 were analyzed retrospectively. The Lasso regression was used to preliminarily screen potential risk factors, which were entered into the multivariable logistic regression analysis to identify preoperative independent risk factors for postoperative AKI. Based on the results, a risk prediction model was developed, and traditional and dynamic nomograms were constructed. The risk prediction model was evaluated using receiver operating characteristic (ROC), calibration curve, and decision curve analysis (DCA).

**Results:**

220 patients (36.2%) developed AKI after surgery. Current smoker, hypertension, heart failure, previous myocardial infarction, cerebrovascular disease, CysC, and NT-proBNP were selected as independent risk factors for AKI. A risk prediction model, a traditional and a dynamic nomogram were developed based on the above factors. The area under the curve (AUC) of the ROC for predicting the risk of postoperative AKI was 0.803 (95% CI 0.769–0.836), with sensitivity and specificity of 84.9% and 63.4%, respectively. The calibration curve slope was close to 1, and the DCA showed that the model produced better clinical benefits when the probability threshold was set at 10%–82%.

**Conclusions:**

We developed a preoperative risk prediction model for AKI after heart valve replacement in patients with normal renal function, which demonstrated satisfactory discrimination and calibration.

## 1 Introduction

Worldwide, approximately 2 million cardiac surgeries are performed annually (1). As one of the common and severe postoperative complications, acute kidney injury (AKI) was reported to occur in 40% of patients undergoing cardiac surgery (2). The occurrence of postoperative AKI, even moderate grades and short duration, not only has a great influence on the prognosis and significantly increases the mortality rate but also prolongs hospitalization, increases monetary costs, and aggravates the burden on patients and their families (3, 4). Patients who undergo heart valve replacement, compared with coronary artery bypass grafting (CABG), are more likely to experience postoperative AKI, for the longer duration of surgery, severer intraoperative hemodynamic fluctuations, and higher proportions of cardiopulmonary bypass (CPB) (5).

Given the lack of effective treatment, the risk prediction and prevention of AKI has become a hot clinical issue that has received extensive attention in recent years (6). Studies have shown that the incidence of postoperative AKI in patients with normal renal function undergoing cardiac surgery ranges from 3.2% to 32.3%, which mainly depends on the definition, criteria, and study population (7, 8). Compared with patients with normal renal function, patients with preoperative renal insufficiency have a higher risk of postoperative AKI. Therefore, previous studies have paid more attention to the prevention of postoperative AKI in patients with renal insufficiency, thus ignoring a wider range of people with normal renal function (9–11). Patients with different baseline renal function seem to have different risk factors for postoperative AKI. However, existing risk prediction models principally focus on the risk assessment of patients with renal insufficiency, which may not apply to patients with normal renal function (12).

Given the above, this study intended to screen preoperative risk factors for AKI in patients with normal renal function after heart valve replacement and establish a preoperative risk prediction model, so as to help clinicians quickly screen high-risk patients early before surgery, then take effective precautions in time, and optimize perioperative anesthesia management to mitigate the risk of postoperative AKI.

## 2 Materials and methods

### 2.1 Study population

This single-center, retrospective observational study was conducted at the Affiliated Hospital of Xuzhou Medical University. The Medical Ethics Committee of the Affiliated Hospital of Xuzhou Medical University approved the study (Jiangsu, China; XYFY2022-KL293-01) and waived informed consent. The trial was registered at ClinicalTrial.gov as ChiCTR2200063711, and the article adhered to the Strengthening the Reporting of Observational Studies in Epidemiology guidelines (STROBE) standards for observational studies (13).

This was a secondary analysis of the data collected from a previous study, which finally included 972 adult patients who underwent cardiac surgery under general anesthesia in the Department of Cardiovascular Surgery, the Affiliated Hospital of Xuzhou Medical University from November 2013 to June 2022 (14). In the present study, we further excluded patients who (1) underwent no heart valve replacement; (2) had a preoperative estimated glomerular filtration rate (eGFR) < 60 ml/(min·1.73 m^2^); (3) underwent emergency surgery.

### 2.2 Outcomes

The primary outcome was any-stage postoperative AKI, which was defined in terms of the Kidney Disease Improving Global Outcomes (KDIGO) classification in 2012 as an absolute increase in postoperative serum creatinine concentration of at least 0.3 mg/dl within 48 h after the surgery or ≥1.5 times baseline within 7 postoperative days (15). The secondary outcomes were as follows: (1) postoperative stage 2 or higher AKI, defined as an increase in serum creatinine of 2.0–2.9 times baseline within 7 postoperative days; (2) postoperative stage 3 AKI, defined as an increase in serum creatinine of at least 4.0 mg/dl or 3.0 times baseline within the initial 7 days, or a new requirement for renal replacement therapy (RRT); (3) postoperative RRT; and (4) in-hospital mortality. Considering the difficulty of accurately measuring urine output in the postoperative period and that urine output is greatly influenced by factors such as blood volume, urine output was not used as a diagnostic indicator for postoperative AKI in this study (16).

### 2.3 Potential confounding variables

Data were collected from the electronic medical record and surgical anesthesia systems, including demographic characteristics [age, gender and body mass index (BMI)], comorbidities (hypertension, heart failure, previous myocardial infarction, atrial fibrillation, pulmonary hypertension, triple vessel disease, cerebrovascular disease, peripheral vascular disease, chronic obstructive pulmonary disease, and diabetes mellitus), left ventricular ejection fraction, history of smoking, previous cardiac surgery, recent interventional operation, preoperative red blood cell transfusion, and laboratory results [serum creatinine, hemoglobin, serum albumin, Cystatin C [CysC], and N-terminal pro-B-type natriuretic peptide [NT-proBNP]]. The estimated glomerular filtration rate was calculated applying the simplified Modification of Diet in Renal Disease (MDRD) formula: eGFR = 186 × Scr^−1.154^ × age^−0.203^ × 0.742 (Female).

### 2.4 Statistical analysis

SPSS statistical software version 26.0 (IBM) and R version 4.2.1 (R Foundation for Statistical Foundation, Vienna, Austria) were used for statistical analysis in this study. Univariable analyses of preoperative baseline characteristics were performed first. Continuous variables were visually assessed for normal distribution using histograms and pp-plots. Continuous variables were reported as means [standard deviations (SDs)] or medians [inter-quartile ranges (IQRs)] and were compared by Student's *t*-tests or Mann–Whitney U-tests; categorical variables were reported as numbers (percentages) and were compared by *χ*^2^ or Fisher's exact tests. In order to mitigate model overfitting and multicollinearity between variables, least absolute shrinkage and selection operator (Lasso) regression was used to screen preoperative potential risk factors preliminarily, and variables with non-zero coefficients were entered into the multivariable logistic regression further to screen preoperative independent risk factors for postoperative AKI. The preoperative risk prediction model was constructed on the basis of the multivariable logistic regression results, and a traditional and a dynamic nomogram were developed, respectively. Multicollinearity between variables was evaluated using the variance inflation factor (VIF): If the VIF was greater than 2, the Pearson correlation matrix was calculated to assess the correlations, and when the Pearson's correlation coefficient among variables was greater than 0.60, only the more critical clinical variable was included in the multivariable logistic regression.

The discriminatory ability of the risk prediction model was evaluated by plotting the receiver operating characteristic (ROC) curve and calculating the area under the curve (AUC). An AUC > 0.70 was considered to show relatively great discrimination. The calibration of the model was assessed by the calibration curve. A 45° diagonal line was deemed to suggest satisfactory calibration. The net benefit of the model was assessed by decision curve analysis (DCA) (17).

Internal validation of the model was performed using the Bootstrap method with the number of samples set at 1,000. A *P*-value less than 0.05 was considered statistically significant. For all analyses, a minimum of 10 events per variable (10EPV) was required to avoid overfitting.

## 3 Results

### 3.1 Preoperative baseline characteristics

**Figure 1:**
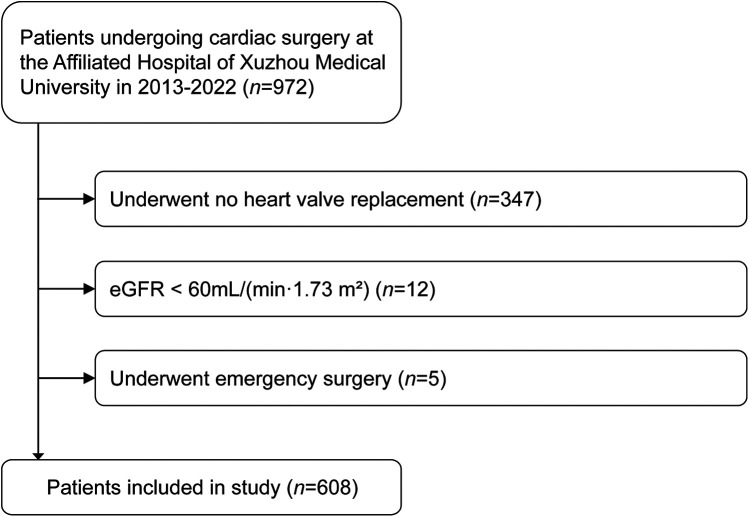
Flow chart presenting patient selection for the study. eGFR, estimated glomerular filtration rate.

According to the inclusion and exclusion criteria, a total of 608 patients undergoing heart valve surgery were included in this study (Figure 1), of which 293 (48.2%) were males and 315 (51.8%) were females. Among eligible subjects, 220 patients (36.2%) experienced postoperative any-stage AKI, including 176 (28.9%) stage 1, 28 (4.6%) stage 2, and 16 (2.6%) stage 3 AKI. 11 (1.8%) patients received RRT, and 29 (4.8%) patients died during their hospital stay. The comparison of preoperative baseline characteristics between patients with and without postoperative AKI is shown in Table 1. Compared to the non-AKI group, patients in the AKI group had a higher incidence of males, current smoker, peripheral vascular disease, hypertension, heart failure, recent interventional operation, triple vessel disease, previous myocardial infarction, cerebrovascular disease, and previous cardiac surgery. Additionally, patients with postoperative AKI had a statistically significant increased age, serum creatinine, CysC, and NT-proBNP, and decreased serum albumin and left ventricular ejection fraction (*P* < 0.05).

**Table 1:** Preoperative baseline characteristics according to the occurrence of postoperative AKI.

Variables	Overall (*n* = 608)	No AKI (*n* = 388, 63.8%)	AKI (*n* = 220, 36.2%)	*P*-value
Age, year, mean (SD)	56.08 (9.84)	54.60 (10.17)	58.68 (8.66)	<0.001
BMI, kg/m^2^, mean (SD)	23.14 (3.34)	22.96 (3.21)	23.45 (3.53)	0.083
Hemoglobin, g/L, mean (SD)	137.13 (18.83)	138.10 (18.92)	135.42 (18.59)	0.093
Preoperative Scr, μmol/L, mean (SD)	68.15 (15.73)	66.48 (13.39)	71.09 (18.86)	<0.001
Serum albumin, g/L, mean (SD)	43.00 (4.62)	43.45 (4.64)	42.19 (4.49)	0.001
LVEF, %, mean (SD)	0.58 [0.52, 0.63]	0.59 [0.53, 0.64]	0.57 [0.49, 0.62]	<0.001
CysC, mg/L, median (IQR)	0.90 [0.78, 1.04]	0.87 [0.77, 0.99]	0.96 [0.82, 1.11]	<0.001
NT-proBNP, pg/ml, median (IQR)	752.00 [374.28, 1,448.50]	638.85 [312.50, 1,036.25]	1,022.00 [542.75, 2,297.00]	<0.001
Male	293 (48.2)	171 (44.1)	122 (55.5)	0.007
Current smoker	115 (18.9)	59 (15.2)	56 (25.5)	0.002
Diabetes mellitus	46 (7.6)	27 (7.0)	19 (8.6)	0.452
Peripheral vascular disease	139 (22.9)	72 (18.6)	67 (30.5)	0.001
Hypertension	153 (25.2)	70 (18.0)	83 (37.7)	<0.001
Recent interventional operation	170 (28.0)	96 (24.7)	74 (33.6)	0.019
Triple vessel disease	15 (2.5)	5 (1.3)	10 (4.5)	0.013
Heart failure	309 (50.8)	148 (38.1)	161 (73.2)	<0.001
Atrial fibrillation	274 (45.1)	172 (44.3)	102 (46.4)	0.628
Previous myocardial infarction	19 (3.1)	5 (1.3)	14 (6.4)	0.001
Pulmonary hypertension	395 (65.0)	250 (64.4)	145 (65.9)	0.714
COPD	20 (3.3)	9 (2.3)	11 (5.0)	0.075
Cerebrovascular disease	274 (45.1)	141 (36.3)	133 (60.5)	<0.001
Preoperative RBC transfusion	6 (1.0)	2 (0.5)	4 (1.8)	0.196
Previous cardiac surgery	45 (7.4)	22 (5.7)	23 (10.5)	0.030

Values are number of patients (%), unless indicated otherwise. AKI, acute kidney injury; SD, standard deviation; IQR, inter-quartile range; BMI, body mass index; Scr, serum creatinine; LVEF, left ventricular ejection fraction; CysC, cystatin C; NT-proBNP, N-terminal pro-B-type natriuretic peptide; COPD, chronic obstructive pulmonary disease; RBC, red blood cell.

### 3.2 Lasso regression for preliminary screening risk factors

**Figure 2:**
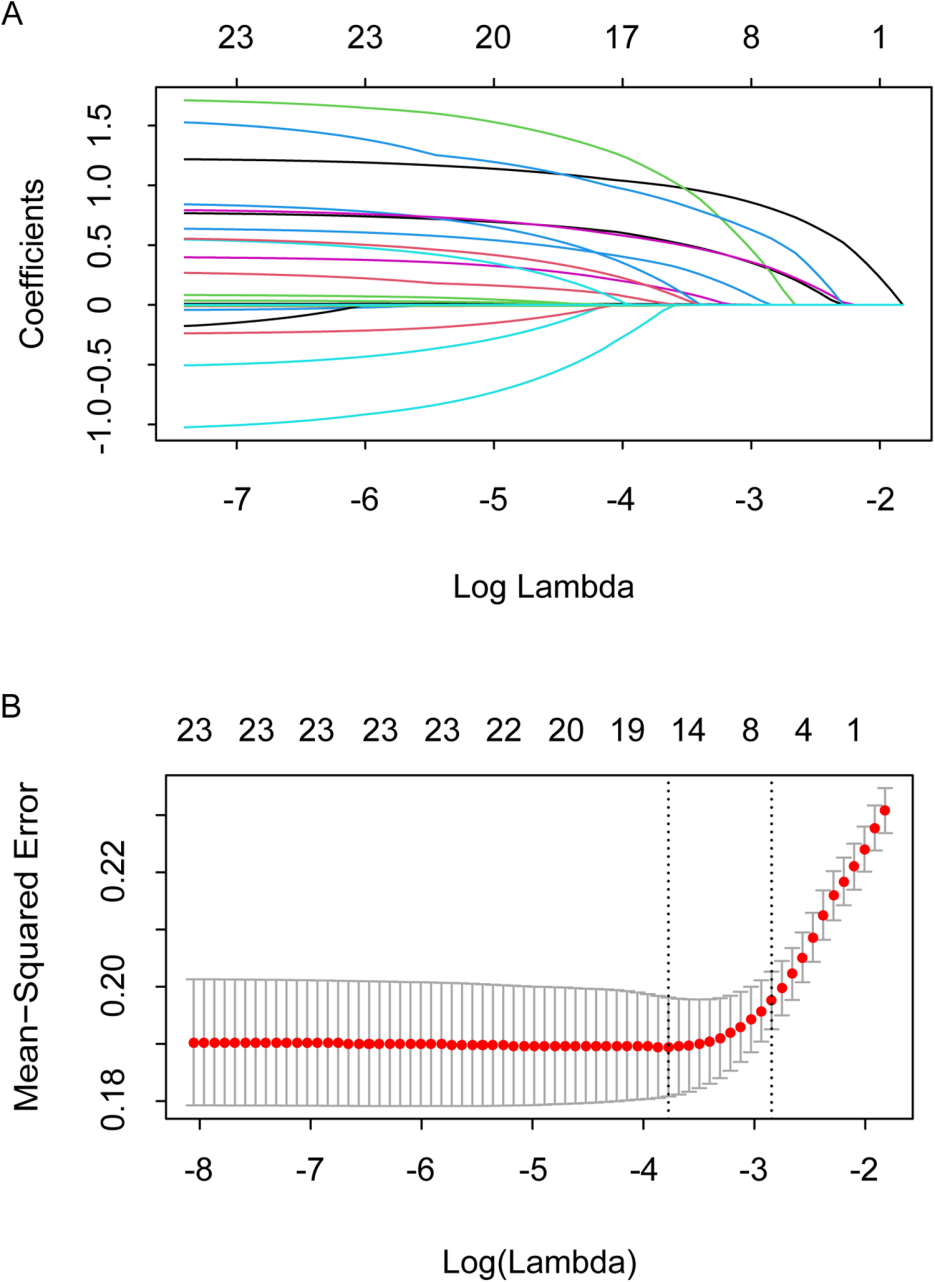
Confounder identification using lasso technique. **(A)** Select the optimal parameter (λ) in the LASSO model using 10-fold cross-validation on the basis of minimum criteria. **(B)** LASSO coefficient profiles of the 23 features. The coefficient profiles are drawn as a function of log (Lambda).

A total of 23 preoperative potential confounding variables were included in the Lasso regression, and 8 potential risk factors for postoperative AKI were preliminarily screened: age, current smoker, hypertension, heart failure, previous myocardial infarction, cerebrovascular disease, CysC, and NT-proBNP (Figure 2).

### 3.3 Establishment of the preoperative risk prediction model

All 8 non-zero characteristic variables selected by the Lasso regression were entered into the multivariable logistic regression full model (Supplementary Table S1). We developed a preoperative risk prediction model based on the results of the multivariable logistic regression, which demonstrated that current smoker [adjusted OR = 2.09, (95% CI: 1.30–3.38), *P* = 0.003], hypertension [adjusted OR = 2.35, (95% CI: 1.52–3.64), *P* < 0.001], heart failure [adjusted OR = 3.42, (95% CI: 2.27–5.21), *P* < 0.001], previous myocardial infarction [adjusted OR = 7.69, (95% CI: 2.50–26.85), *P* < 0.001], cerebrovascular disease [adjusted OR = 2.33, (95% CI: 1.58–3.44), *P* < 0.001], CysC [adjusted OR = 1.16, (95% CI: 1.04–1.29), *P* = 0.007], and NT-proBNP [adjusted OR = 1.27, (95% CI: 1.10–1.46), *P* = 0.001] were preoperative independent risk factors for postoperative AKI in patients with normal renal function, whereas no statistically significant associations were observed with age (Table 2). The variance inflation factors of all the variables in the model were less than 2, suggesting that the multicollinearity among the variables was small.

**Table 2:** Preoperative risk prediction model for postoperative acute kidney injury after heart valve replacement in patients with normal renal function.

Risk factor	β-coefficient	Adjusted OR (95% CI)	*P*-value
CysC^a^	0.146	1.16 (1.04–1.29)	0.007
NT-proBNP^b^	0.236	1.27 (1.10–1.46)	0.001
Current smoker	0.738	2.09 (1.30–3.38)	0.003
Cerebrovascular disease	0.844	2.33 (1.58–3.44)	<0.001
Hypertension	0.853	2.35 (1.52–3.64)	<0.001
Heart failure	1.231	3.42 (2.27–5.21)	<0.001
Previous myocardial infarction	2.039	7.69 (2.50–26.85)	<0.001

^a^ Per 0.1 mg/L increase in CysC.

^b^ Per 1,000 pg/ml increase in NT-proBNP. OR, odds ratio; CI, confidence interval; CysC, cystatin C; NT-proBNP, N-terminal pro-B-type natriuretic peptide.

### 3.4 Construction of traditional and dynamic nomograms

**Figure 3:**
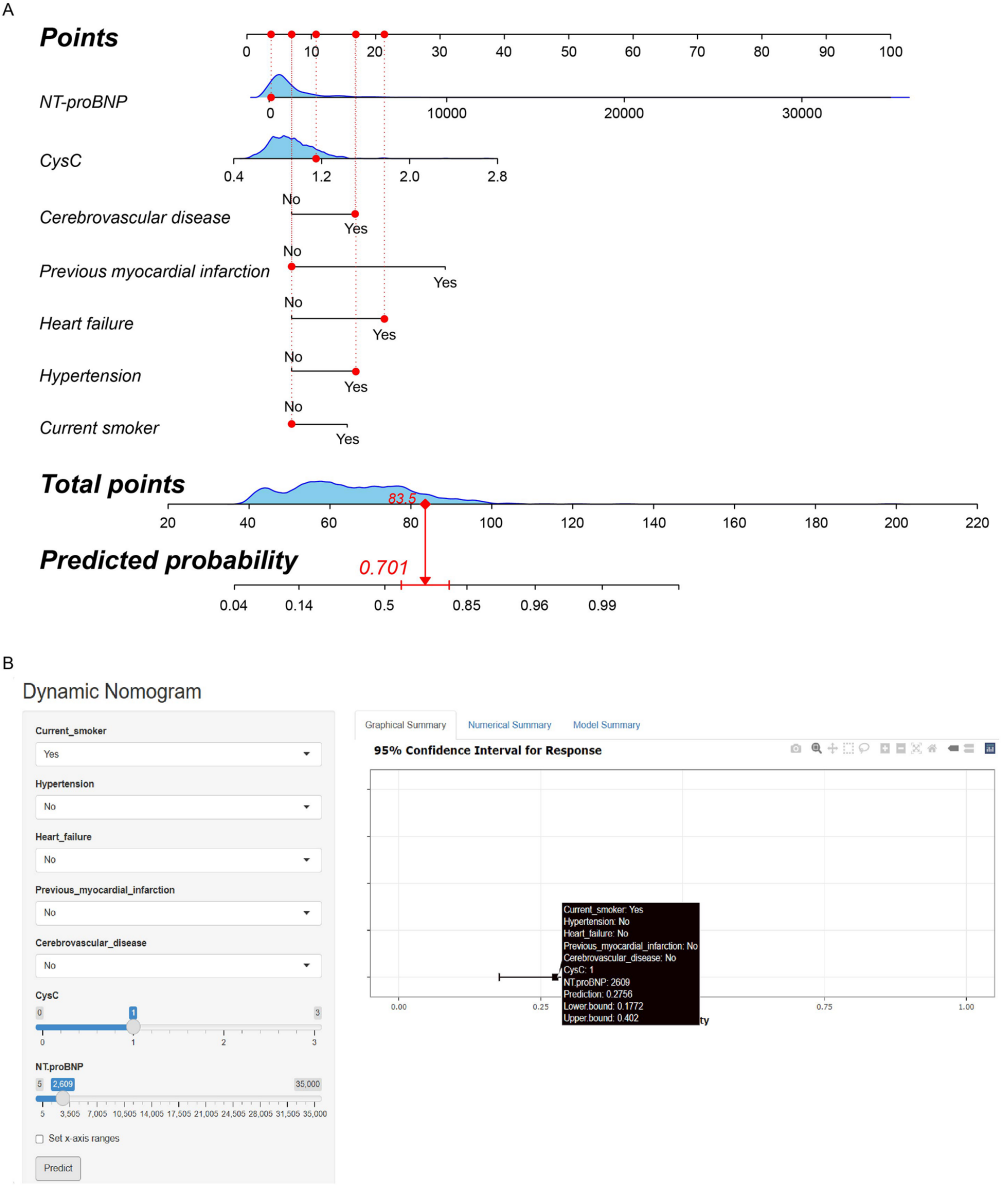
**(A)** Traditional nomogram of the AKI risk prediction model after heart valve replacement in patients with normal renal function. For instance, a patient was a current non-smoker, with preoperative NT-proBNP 112 pg/ml, CysC 1.15 mg/L, cerebrovascular disease, heart failure, and hypertension, without previous myocardial infarction. The total point was calculated as 83.5, which corresponded to the probability of 70.1%. **(B)** Dynamic nomogram of the AKI risk prediction model after heart valve replacement in patients with normal renal function. For instance, a patient was a current smoker, with preoperative NT-proBNP 2,609 pg/ml, CysC 1.00 mg/L, without hypertension, heart failure, previous myocardial infarction and cerebrovascular disease. The web page demonstrated that the probability of postoperative AKI was 27.6% (95% CI: 17.7%–40.2%).

A traditional nomogram was constructed based on these 7 preoperative risk factors for predicting AKI after heart valve replacement in patients with normal renal function. As shown in Figure 3A, a vertical line was drawn from the axis of each risk factor to the “Points” axis to obtain the point for each risk factor, and all points were summed to obtain the total points and the corresponding probability of postoperative AKI.

Considering the difficulty of application in clinical practice, we subsequently constructed a user-friendly and online dynamic nomogram of the risk prediction model (Figure 3B). The dynamic nomogram is displayed at https://aki-model.shinyapps.io/dynnomapp/. After entering the values of these 7 risk factors, the web page will demonstrate the probability of postoperative AKI, the 95% confidence interval, and the parameters of the prediction model. For instance, in patients with preoperative NT-proBNP 2,609 pg/ml, CysC 1.00 mg/L, and current smoker, without hypertension, heart failure, previous myocardial infarction, and cerebrovascular disease, 27.6% (95% CI: 17.7%–40.2%) of them would experience postoperative AKI.

### 3.5 Evaluation of the risk prediction model

**Figure 4:**
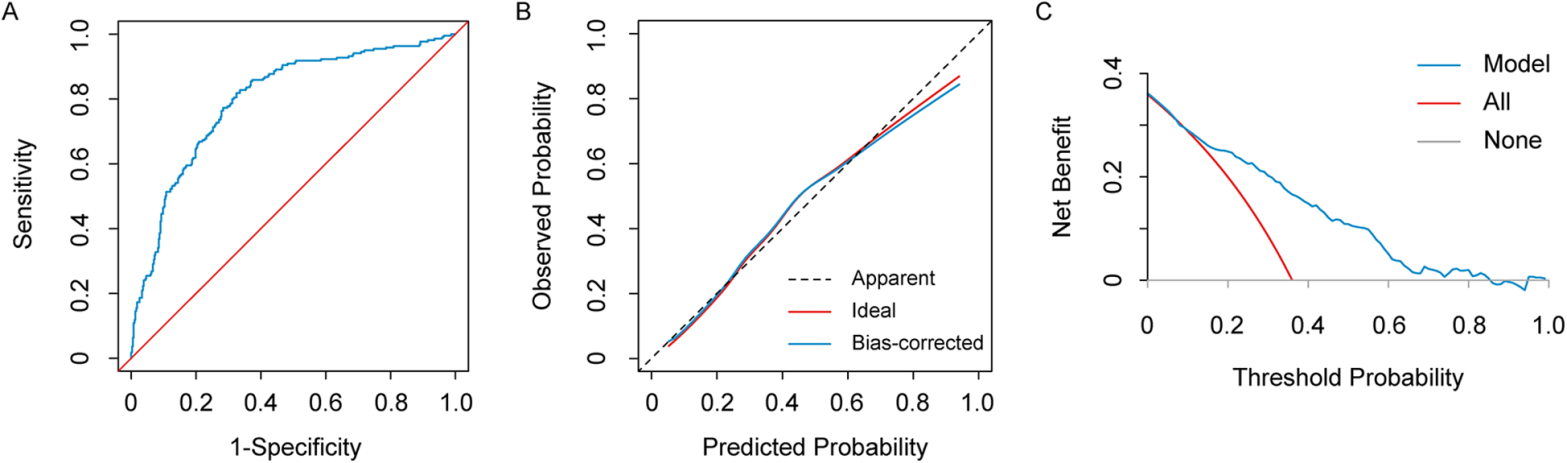
The receiver operating characteristic (ROC) curve **(A)**, calibration curve **(B)**, and decision curve analysis (DCA) **(C)** of the preoperative risk prediction model for AKI after heart valve replacement in patients with normal renal function.

The ROC curve of this model for predicting the risk of AKI after heart valve replacement in patients with normal renal function showed an AUC of 0.798 (95% CI: 0.762–0.835), a sensitivity of 81.8%, a specificity of 67.5%, which demonstrated satisfactory discrimination (Figure 4A). The slope of the plotted model calibration curve was close to 1, which indicated great agreement between the predictions and observations, suggesting the satisfactory calibration of the model (Figure 4B). The decision curve analysis showed that the prediction model was better than all and none curves at threshold probabilities ranging from 10% to 82% (Figure 4C). These results suggested that the model we developed provided a higher net benefit over a reasonably wide range of threshold probabilities for predicting postoperative AKI and, therefore, has excellent clinical utility. In the bootstrapping internal validation, we sampled the data 1,000 times and obtained an AUC of 0.789, which was similar to this prediction model, suggesting that the model was robust.

## 4 Discussion

The occurrence of postoperative AKI significantly elevates the incidence of in-hospital death and other complications, affects long-term prognosis, and increases the economic burden on patients (18). In this study, a total of 220 patients developed AKI in the 608 patients who underwent heart valve replacement, with an incidence of 36.2%, which is similar to previous studies (2, 9, 19). In spite of great advances in the diagnosis, surgery, and anesthetic management of AKI in recent years, the incidence of postoperative AKI has remained essentially unchanged. The results of this study demonstrated that current smoker, hypertension, heart failure, previous myocardial infarction, cerebrovascular disease, CysC, and NT-proBNP are preoperative independent risk factors for AKI after heart valve replacement in patients with normal renal function.

Wang et al. found that in a study involving 35,337 patients who underwent cardiac surgery, 16,438 (46.5%) had a history of smoking, which was a significant independent risk factor for postoperative AKI (9). Similarly, Birnie et al. concluded that patients with smoking history were at an increased risk of postoperative AKI (5). In addition, the risk of postoperative AKI was significantly higher in patients who had not quit smoking than in those who had quit. Our study also confirmed that current smoker is one of the preoperative risk factors for AKI. However, we did not further investigate the effect of quitting smoke before surgery on AKI. In recent years, researchers have established a variety of clinical prediction models to estimate the risk of AKI after cardiac surgery, among which Ngu et al. and Zhao et al. have verified that the risk of postoperative AKI was significantly higher in patients with heart failure, and that heart failure was independently associated with postoperative AKI (20, 21). Obviously, decreased cardiac output in patients with heart failure causes renal ischemia and hypoxia, and reduces glomerular filtration rate, exacerbating renal injury. Numerous studies have shown that patients with hypertension were more prone to postoperative AKI. Similarly, in this study, the proportion of patients with a history of hypertension was higher in those who developed postoperative AKI than in those who did not, with the mechanism possibly related to renal parenchymal ischemia and nephron reduction caused by prolonged and sustained hypertension (22, 23). A few studies have suggested that postoperative AKI was associated with previous myocardial infarction and cerebrovascular disease, and our present study reconfirmed the correlation (21, 24). Patients with previous myocardial infarction and cerebrovascular disease often have insufficient perfusion of cardiovascular, cerebrovascular, as well as kidney before surgery. Even if the preoperative renal function tests are normal, the renal reserve and compensatory capacity are still impaired, making the renal function more susceptible to damage than that of normal people. As a sensitive biomarker that reflects changes in glomerular filtration rate to a certain extent, CysC has been confirmed by several studies to be used in the early diagnosis of AKI with good predictive performance. Studies have found that patients with postoperative AKI demonstrated significantly higher preoperative CysC than those without AKI, and CysC was associated with increased odds of AKI in a dose-dependent manner (2). Elevated levels of NT-proBNP are significantly associated with an increased risk of postoperative cardiovascular complications (including myocardial infarction, heart failure, arrhythmia, cardiogenic shock, etc.) and death. Additionally, it has also been confirmed as an independent predictor of postoperative AKI, and its ability to predict AKI has been widely discussed and acknowledged. Wang et al. found that the inclusion of NT-proBNP in their prediction model for AKI after cardiac surgery improved the model's predictive ability by 24% (9). The possible mechanism is that elevated preoperative NT-proBNP levels indicate cardiovascular dysfunction and impaired hemodynamics, which promotes the progression of AKI.

The prediction model established in this study incorporates biomarkers CysC and NT proBNP, which allowed for better prediction of AKI risk and improved the predictive ability of the model. In recent years, several inflammatory biomarkers have been confirmed as indicators for predicting the risk of AKI. Neopterin, a non-specific indicator of cellular immunity, is produced by activated macrophages as a response to inflammation and immune system activation (19, 25). In patients undergoing on-pump cardiac surgery, postoperative AKI was observed to be independently associated with increased preoperative neopterin, which was a novel and effective marker for the preoperative prediction of AKI (19). In addition, soluble urokinase plasminogen activator receptor (suPAR) and neutrophil gelatinase-associated lipocalin (NGAL), which were also related to inflammation and immune response, have been found to have the highly predictive nature for diagnosis of AKI after cardiac surgery (26, 27). The inclusion of these inflammatory biomarkers in the clinical prediction model for AKI has the potential to enhance the predictive capability for identifying individuals at risk of developing postoperative AKI.

The risk prediction model developed in this study only analyzed preoperative risk factors and did not adjust for the other potential intraoperative and postoperative risk factors, such as cumulative duration of intraoperative hypotension, duration of CPB, and vasopressin dose, which were not available preoperatively. These intraoperative and postoperative variables may influence the risk of postoperative AKI to some extent, and the predictive performance of the model may be further improved by incorporating them. However, our goal is to rapidly screen high-risk patients early in the preoperative period in order to take precautions, such as avoiding prolonged severe hypotension, and thus to improve the clinical utility of the risk prediction model. We have verified that the risk prediction model has satisfactory discrimination, calibration, and clinical utility. Even though intraoperative and postoperative factors play an essential role in the development of AKI, the risk of postoperative AKI can still be accurately predicted before surgery, which may be due to the correlations between preoperative risk factors and intraoperative and postoperative factors. For example, hypertension and heart failure are suitable surrogate variables for severe and prolonged hypotension and other relevant intraoperative and postoperative factors (20, 28).

Previous research has demonstrated that the incidence of AKI after heart valve replacement reached up to 33.24%, and the mortality rate among patients who developed postoperative AKI was 6.49% (29). Compared with patients undergoing valve surgery, patients undergoing CABG have a lower incidence of AKI, amounting to 19.0% (30). Heart valve replacement has been verified as an independent risk factor of AKI after cardiac surgery (30, 31). We supposed that patients undergoing heart valve replacement are more likely to have multiple preoperative underlying diseases, poor heart function, prolonged duration of surgery and CPB, and to experience severe intraoperative hypotension, which lead to the high risk of postoperative AKI. We speculated that the perioperative risk factors for AKI vary by different types of surgery. Therefore, we conducted this research to analyze the risk factors of AKI among heart valve replacement patients. In the future, further in-depth research on other types of cardiac surgery will be conducted to explore whether the prediction model we built could be extrapolated to a broader cardiac surgery population.

This study has some limitations. On the one hand, this is a retrospective observational study. Although we included as many confounders as possible, there were still some residual confounders that were not adjusted for, which may affect the accuracy of the study results. On the other hand, this is a single-center study, and no external validation has been conducted. Although the internal validation has suggested that the risk prediction model had satisfactory predictive performance, its extrapolation capability still needs to be tested. Next, more patients need to be recruited to confirm the reproducibility of this risk prediction model and to lay the foundation for its clinical application. Moreover, in this study, we included preoperative continuous variables such as age, BMI, and CysC in the multivariable analysis, and did not convert them to categorical variables for further analysis. Although the use of continuous variables could avoid the loss of information, the clinical utility of the risk prediction model needs to be improved.

## 5 Conclusions

In summary, the incidence of AKI after heart valve replacement in patients with normal renal function was 36.2%. Current smoker, hypertension, heart failure, previous myocardial infarction, cerebrovascular disease, CysC, and NT-proBNP were independent risk factors. The preoperative risk prediction model developed in this study can effectively predict the risk of AKI after heart valve replacement in patients with normal renal function, facilitating early screening of high-risk patients and timely adoption of preventive measures to reduce the incidence of postoperative AKI.

## Data Availability

The raw data supporting the conclusions of this article will be made available by the authors, without undue reservation.
