# Supplementary material for: Risk factors analysis and prediction model establishment of acute kidney injury after heart valve replacement in patients with normal renal function
Source: Front Cardiovasc Med. 2025 Feb 10;12:1422870. doi: 10.3389/fcvm.2025.1422870 (PMC11847868; doi:10.3389/fcvm.2025.1422870)
Supplement: Supplementary file 1 [file Table1.pdf]

Supplementary Table 1. Multivariable logistic regression full model. OR, odds ratio; CI, confidence interval; CysC, Cystatin C; NT-proBNP, N-terminal pro-B-type natriuretic peptide. [a] Per 0.1 mg/L increase in CysC. [b] Per 1000 pg/ml increase in NT-proBNP.

**Risk factor**	**β-coefficient**	**Adjusted OR (95% CI)**	***P*-value**
Age	0.012	1.01 (0.99-1.04)	0.287
CysC [a]	0.134	1.14 (1.03-1.28)	0.015
NT-proBNP [b]	0.229	1.26 (1.09-1.45)	0.002
Current smoker	0.752	2.12 (1.31-3.44)	0.002
Cerebrovascular disease	0.787	2.20 (1.47-3.29)	<0.001
Hypertension	0.805	2.24 (1.44-3.49)	<0.001
Heart failure	1.230	3.42 (2.27-5.21)	<0.001
Previous myocardial infarction	2.037	7.67 (2.49-26.90)	<0.001
